# Hierarchical ZnO/NiCo_2_O_4_/Nb_2_C: A Novel Counter Electrode for Platinum-Free Dye-Sensitized Solar Cells

**DOI:** 10.1021/acsomega.6c07351

**Published:** 2026-09-07

**Authors:** Nizamudeen Cherupurakal, Ramachandran Krishnapriya, Saifudeen Kabeer, Sambasivam Sangaraju

**Affiliations:** † District 4.0 Project, Science and Innovation Park, 11239 United Arab Emirate University, Al Ain, P.O. Box 15551, UAE; ‡ Associate Provost for Research Office, United Arab Emirates University, Al Ain, P.O. Box 15551, UAE; § National Water and Energy Center, United Arab Emirates University, Al Ain, P.O. Box 15551, UAE

## Abstract

The development of sustainable, cost-effective, and high-performance counter electrodes (CEs) remains a major challenge in dye-sensitized solar cell (DSSC) research. Although Pt remains the benchmark CE because of its excellent electrocatalytic activity and electrical conductivity, its scarcity and high cost motivate the development of efficient Pt-free alternatives. Herein, we report a novel Pt-free counter electrode based on a MOF-derived ZnO/NiCo_2_O_4_/Nb_2_C MXene composite for DSSC applications. The counter electrode materials were synthesized by a hydrothermal method and systematically characterized to evaluate their structural, morphological, and photovoltaic properties. X-ray diffraction analysis revealed characteristic peaks at 36.3°, 34.1°, and 40.3°, confirming the formation of ZnO, NiCo_2_O_4_, and Nb_2_C. The optimized ZnO/NiCo_2_O_4_/Nb_2_C MXene counter electrode achieved a power conversion efficiency (PCE) of 8.08%, comparable to that of Pt-based DSSCs under standard illumination. This enhanced performance is attributed to the synergistic effects of high catalytic activity, rapid charge transfer, and robust structural integrity in the MOF-derived ZnO/NiCo_2_O_4_/Nb_2_C MXene composite. Also, the addition of Nb_2_C MXene significantly enhanced the overall electrical conductivity and accelerated electron transfer kinetics at the electrolyte–electrode interface. These findings demonstrate that the ZnO/NiCo_2_O_4_/Nb_2_C MXene composite is a promising platinum-free counter electrode for advancing DSSCs toward practical commercialization and large-scale energy harvesting applications.

## Graphical abstract

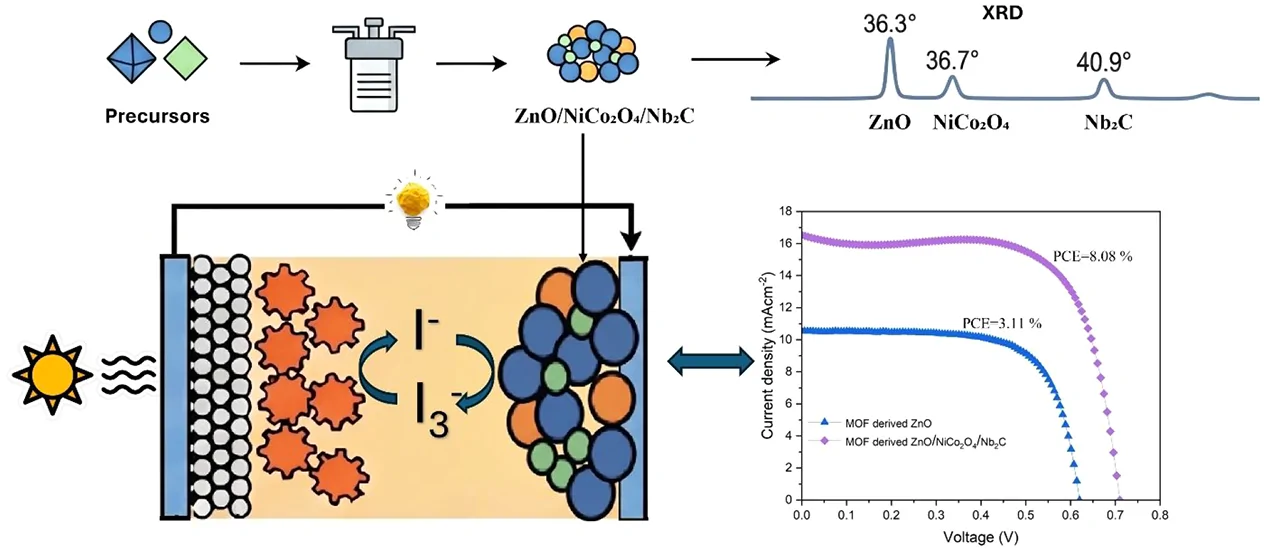

## 1 Introduction

Dye-sensitized solar cells, widely recognized as third-generation photovoltaic devices, offer distinctive advantages, including facile fabrication, transparency, flexibility, and adaptability, making them strong candidates for sustainable solar energy conversion.^1–5^ DSSCs rely on abundant, low-toxicity materials, simple fabrication, and low processing temperatures, making them suitable for affordable and eco-friendly solar energy conversion and viable candidates for industrial-scale energy solutions.^6,7^ Despite their inherent advantages, DSSCs face critical challenges such as their comparatively lower power conversion efficiency (PCE) relative to silicon-based solar cells and the sustained requirement to enhance long-term operational stability. Current research efforts aim to address these limitations through material innovations and device optimization.

The components of a DSSC comprise a conductive substrate, a semiconductor anode, a sensitizing dye, an iodine-based electrolyte, and a counter electrode.^8,9^ Here, the counter electrode (CE) is a crucial component of dye-sensitized solar cells (DSSCs), accountable for collecting electrons from the external circuit and catalyzing the reduction of triiodide (I_3_^–^) to iodide (I^–^) in the electrolyte, thereby completing the electrical circuit and facilitating the regeneration of the redox mediator.^10,11^ Platinum (Pt) is the most widely used material for CEs due to its outstanding catalytic activity and high conductivity.^10,12^ However, its high cost, scarcity, and limited stability in corrosive electrolytes restrict its large-scale application and have motivated researchers to focus on exploring earth-abundant, low-cost, and highly active substituents.^13–15^ As an efficient replacement for Pt, a range of materials implemented so far includes carbon-based materials, metal sulfides, metal oxides, conducting polymers, and different hybrid composites.^16–20^ Recent studies have focused on hybrids because they integrate the complementary advantages of individual components, combining high conductivity, abundant catalytic sites, and hierarchical porous sites, thereby achieving superior electrocatalytic activity, faster charge transfer kinetics, and improved long-term stability compared to single-component counterparts.^21,22^

Metal–organic frameworks (MOFs) and their derivatives have emerged as promising alternatives to platinum (Pt) for counter electrodes (CEs) in dye-sensitized solar cells (DSSCs), owing to their unique structural features, high surface area, tunable porosity, and catalytic activity. The use of MOF-derived materials addresses the high cost and limited stability of traditional Pt electrodes, offering potential for high efficiency and low-cost DSSCs.^23,24^ Carbon materials derived from ZIF-8 (a zeolitic imidazolate framework) have been applied as CEs in DSSCs, demonstrating easy fabrication, large specific surface area, and high catalytic activity for the reduction of I_3_^–^ ions. These electrodes achieved a power conversion efficiency (PCE) of 7.32%, similar to Pt-based cells.^23^ N-doped carbon sheets synthesized from ZIF-8/graphene oxide composites exhibited superior electrocatalytic activity for I_3_^–^ reduction, achieving a PCE of 8.2%, which surpasses that of Pt CEs (7.6%).^25^ Moreover, MOF-5, when combined with a conductive polymer binder (s-PT) and deposited on carbon cloth, forms a hierarchical electron transfer network. This composite CE achieved a PCE of 8.91%, and under dim-light conditions, efficiency reached 9.75%.^26^ However, direct carbonization of MOFs can lead to disintegration and aggregation, reducing electronic conductivity, which is one of the main concerns in integrating MOF-derived CEs.^27^

Emerging materials such as NiCo_2_O_4_ and NiCo_2_S_4_ were also found to be used as counter electrodes for various applications. Recent studies show that NiCo_2_O_4_/ NiCo_2_S_4_ based electrodes deliver high catalytic activity toward I^–^/I_3_^–^ reduction, good conductivity, and significant cost advantages over Pt. However, to maximize performance, NiCo_2_O_4_/ NiCo_2_S_4_ was used in composites along with materials such as carbon allotropes and metal oxides.^28–30^ Li et al. used NiCo_2_O_4_ with electrospun carbon nanofibers (CNFs) as CEs in DSSCs and reported an efficiency of 7.80%.^31^ Similarly, core–shell nanostructures of NiCo_2_O_4_@MnMoO_4_ were used as CEs and reported up to 11.87% efficiency.^32^ Nukunudompanich et al. implemented NiCo_2_O_4_@ZnS nanorods with MWCNTs as CEs, and an efficiency of 10.03% was reported.^33^ It can be seen that mixing NiCo_2_O_4_ with a conducting material enhances the overall efficiency of DSSCs. MXenes are also known for exceptional conductivity along with high hydrophilicity and mechanical strength. MXene-based composites have reported superior electrocatalytic activity and lower charge-transfer resistance, often used as CEs, and the PCEs match or surpass Pt.^34^ Hu et al. used CNTs/Ti_3_C_2_T_*x*_ MXenes composite CEs for DSSCs. They showed low charge-transfer resistance and good electrocatalytic activity in I^–^/I_3_^–^ electrolyte. The PCE was very close to Pt, 5.83%. Similarly, MXene-graphene was used by Nagalingam et al. and reported a PCE of 6.4%.^35^ Furthermore, MXene/NiCo_2_S_4_ 2D/3D heterostructure CEs were used, and 8.76% efficiency was achieved.^36^

From the literature, it can be seen that most studies use binary composite, and MOF-derived materials and Nb_2_C MXenes have not been well explored as CEs in DSSCs. In this study, we report a novel MOF-derived ZnO/NiCo_2_O_4_/Nb_2_C tertiary hybrid as a counter-electrode material for DSSCs. The synthesis was carried out in a hydrothermal system. The materials obtained were confirmed using XRD and FTIR. The morphological analysis was conducted using FESEM and HRTEM. The synergistic integration of MOF-derived ZnO and NiCo_2_O_4_ within an Nb_2_C MXene matrix will create a multi-component hybrid architecture that significantly lowers the charge-transfer resistance for the I^–^/I_3_^–^ redox reaction. This structure provides a high density of active sites and accelerated electron pathways, leading to photovoltaic conversion efficiencies in DSSCs that are comparable to or exceed traditional Pt-based counter electrodes while offering superior long-term electrochemical stability.

## 2 Materials and Methods

Zinc nitrate hexahydrate (Zn­(NO_3_)_2_.6H_2_O), 2-methylimidazole, nickel­(II) nitrate hexahydrate (Ni­(NO_3_)_2_.6H_2_O), cobalt­(II) nitrate hexahydrate (Co­(NO_3_)_2_.6H_2_O), urea, *N*-methyl-2-pyrrolidone (NMP), polyvinylidene fluoride (PVDF), and N719 dye were purchased from Sigma-Aldrich (USA) and used without further purification. The hydrothermal process was carried out using deionized water in a BAOSHISHAN 100 mL autoclave with a PTFE liner.

### 2.1 Synthesis of MOF-Derived ZnO

ZIF-8 was synthesized following the method reported by Mani et al.^37^ Typically, solution A comprising Zn­(NO_3_)_2_·6H_2_O (2.376 g) in 40 mL of DI water was added dropwise to solution B, containing 2-methylimidazole (1.312 g) in 40 mL of DI water. The mixture was stirred for 4 h, after which the ZIF-8 precipitate was isolated via centrifugation and washed repeatedly with methanol and DI water. The product was dried at 70 °C for 12 h. Finally, MOF-derived ZnO was obtained by calcining the ZIF-8 crystals at 500 °C for 5 h in air.

### 2.2 Synthesis of MOF-Derived ZnO/NiCo_2_O_4_/Nb_2_C Hybrid

The ZnO/NiCo_2_O_4_/Nb_2_C hybrid was synthesized via a hydrothermal method followed by thermal annealing. Initially, Ni­(NO_3_)_2_·6H_2_O (0.43 g, 0.03 M), Co­(NO_3_)_2_·6H_2_O (1.1 g, 0.12 M), and urea (1.5 g, 0.5 M) were dissolved in 50 mL of double-distilled water (DDW) with stirring for 30 min. In a separate vessel, 1 g of MOF-derived ZnO and 0.56 g of Nb_2_C were dispersed in 30 mL of DDW. The metal salt solution was added dropwise to the dispersion and stirred for 45 min, followed by 15 min of bath sonication to ensure homogeneity. The mixture was then sealed in a 100 mL Teflon-lined stainless-steel autoclave and maintained at 120 °C for 6 h. The resulting precipitate was collected, washed thoroughly with DDW and ethanol, and dried overnight at 80 °C. Finally, the hybrid material was obtained by annealing the dried powder at 450 °C for 2 h.

### 2.3 Fabrication of DSSCs

The DSSC devices were fabricated according to a previously reported procedure.^8^ To prepare the photoanode, TiO_2_ was ground with four drops of *N*-methyl-2-pyrrolidone (NMP) for 25 min to produce a homogeneous, viscous slurry. This slurry was coated onto a fluorine-doped tin oxide (FTO) substrate and dried at 80 °C overnight. The resulting film was sensitized by immersion in a 3 mM N719 dye solution for 24 h. Separately, the counter electrode (CE) was prepared by mixing 95 mg of the synthesized CE material with 5 mg of polyvinylidene fluoride (PVDF) and NMP. This mixture was uniformly deposited onto an FTO glass substrate and dried at 80 °C for 8 h. Finally, the photoanode and CE were sandwiched together with a separator, and an iodide/triiodide (I^–^/I_3_^–^) electrolyte was introduced into the active area. The photovoltaic parameters, including fill factor (FF) and power conversion efficiency (PCE), were calculated using eqs 1 and 2.

1 $$\mathrm{FF}=\frac{V_{mp}\times J_{mp}}{V_{oc}\times J_{sc}}$$

2 $$\mathrm{PCE}\left(\%\right)=\left(\mathrm{FF}\times J_{sc}\times V_{oc}\right)/P_{in}$$

### 2.4 Characterization

The structural properties of the synthesized materials were investigated via X-ray diffraction (XRD) using a Panalytical-Empyrean diffractometer (Netherlands) equipped with Cu Kα radiation (λ = 1.54 Å) operated at 45 kV and 40 mA; diffraction patterns were recorded across a 2θ range of 10°–80°, with phase identification performed against standard ICDD database patterns. Functional group analysis was conducted through Fourier-transform infrared (FTIR) spectroscopy in ATR mode using a Shimadzu IRSpirit-X within the 400–4000 cm^–1^ range. Optical properties were evaluated using a Varian Cary 4000 UV–visible spectrophotometer (200–800 nm) and photoluminescence (PL) spectroscopy (430–650 nm). The surface elemental composition and corresponding electronic valence states were analyzed using an Omicron Nanotechnology (Oxford Instruments) X-ray photoelectron spectrometer, utilizing a monochromatic Al Kα X-ray source. The spectrum was fitted employing a Gaussian functions, and peak correction were made. For device evaluation, the photovoltaic performance and electrochemical impedance spectroscopy (EIS) of the masked DSSCs were measured under AM 1.5G illumination (one sun) using a PET Photo Emission Tech SS50AAA solar simulator and a CHI660e electrochemical workstation (CH Instruments), respectively. Cyclic voltammetry was recorded in the potential range of −0.1 V to 1 V. EIS data were acquired from 1 Hz to 0.1 MHz with a 10 mV AC amplitude and subsequently analyzed using Z-fit software to correlate the interfacial charge-transfer dynamics with device efficiency.

## 3 Results and Discussion

X-ray diffraction (XRD) analysis was performed to determine the material formation, phase composition, and purity of the synthesized MOF-derived ZnO, NiCo_2_O_4_, Nb_2_C MXene, binary (ZnO/NiCo_2_O_4_) and the ternary (ZnO/NiCo_2_O_4_/Nb_2_C) composites. The diffraction patterns for all synthesized materials are shown in Figure 1a. For the MOF-derived ZnO, sharp and well-defined peaks were observed at 2θ values of 31.4° and 36.3°, which are indexed to the (110) and (101) lattice planes of phase-pure wurtzite ZnO.^38,39^ These peaks correspond to a hexagonal crystal structure, showing excellent agreement with the standard JCPDS card no. 036–1451. The broadness and moderate intensity of the obtained peaks suggest nanosized crystal formation, confirming the MOF-derived nanostructured oxide ZnO. For NiCo_2_O_4_, the characteristic peaks were found at 31.1°, 36.7°, and 44.6°, corresponding to the planes (220), (311), and (222) of the fcc cubic spinel structure.^40–42^ No other impurity peaks were observed, confirming phase-pure crystalline NiCo_2_O_4_. Similarly, for the Nb_2_C MXene, the obtained peaks at 34.9° and 40.9° correspond to the (111) and (200) planes of the distinct cubic/fcc-like carbide diffraction profile. In the binary and tertiary composites, the peaks remain consistent with those of the pristine materials, with no observable shifts. This indicates that the structural frameworks of the individual components are preserved during composite synthesis, suggesting that the constituents are effectively integrated without inducing significant lattice distortions or chemical interactions that would alter the crystal structures. Furthermore, the absence of additional peaks confirms the high phase purity of the final composite, verifying that no secondary impurities were formed during the fabrication process. XRD results confirm the phase-pure ZnO/NiCo_2_O_4_/Nb_2_C heterostructure formation with preserved crystallinity and interfacial coupling among the constituent phases, which are expected to be beneficial for charge transport and electrochemical performance.

**Figure 1:**
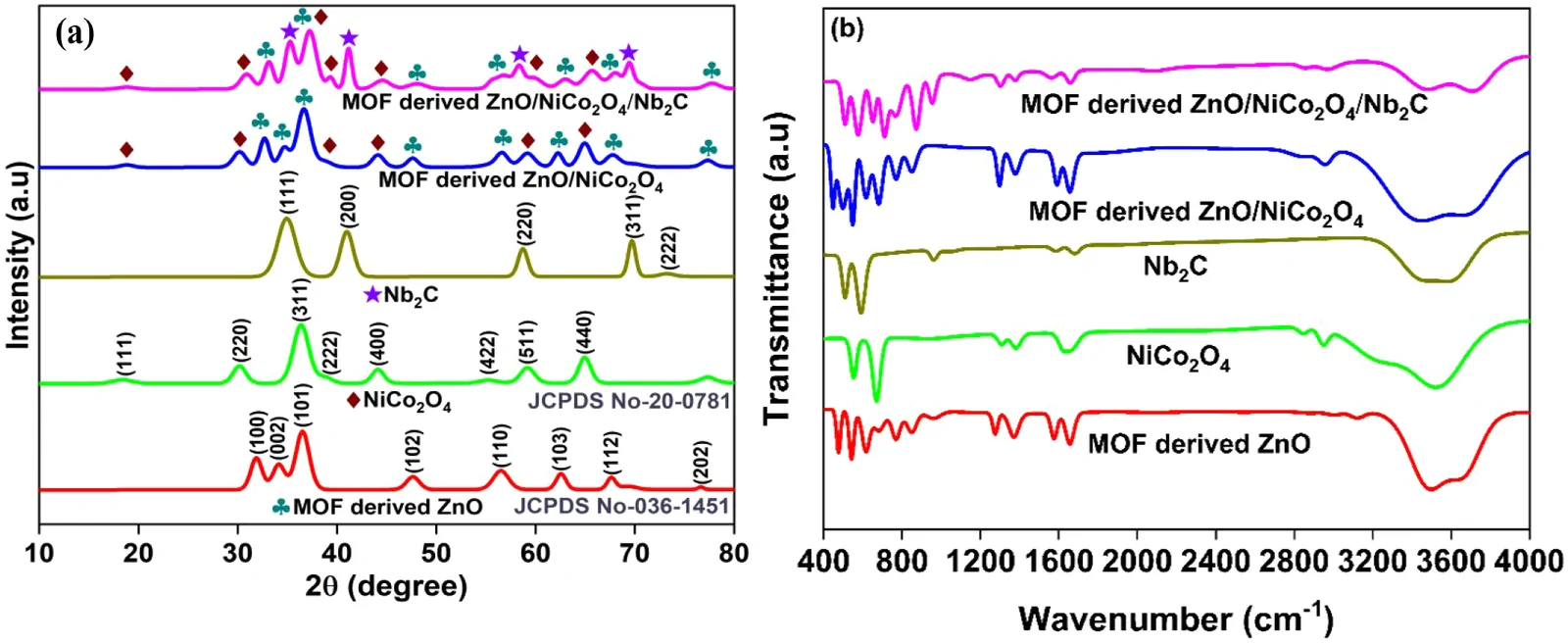
(a) X-ray diffraction patterns of the synthesized CE materials; (b) FTIR spectrum of the synthesized CE materials.

The crystallite size (*D*), microstrain (ε), and dislocation density (δ) of the synthesized materials were calculated using the standard eqs 3–5 and are summarized in Table 1.

3 $$D=\frac{k\lambda }{\beta \;\cos\theta }$$

4 $$\varepsilon =\frac{\beta }{4\;\tan\theta }$$

5 $$\delta =\frac{1}{D^{2}}$$

where *k* is the Scherrer constant (0.9), λ is the X-ray wavelength (1.5406 Å), β is the full width at half maximum (FWHM) of the diffraction peaks, and θ is the Bragg diffraction angle.

**Table 1:** Crystallite Size (*D*), Microstrain (ε), and Dislocation Density (δ) of the Synthesized Materials

**Materials**	**Crystallite Size *D* (nm)**	**Microstrain (ε) × 10** ^**–3**^	**Dislocation Density (δ) × 10** ^**–4**^
ZnO	17.10	6.76	34.21
NiCo_2_O_4_	13.65	8.49	53.69
Nb_2_C	11.60	10.40	74.34
ZnO/NiCo_2_O_4_	10.48	10.99	91.04
ZnO/NiCo_2_O_4_/Nb_2_C	7.94	14.52	158.44

The pristine materials ZnO, NiCo_2_O_4_, and Nb_2_C exhibit larger crystallite sizes of 17.10, 13.65, and 11.60 nm, respectively. Upon forming the binary hybrid, the crystallite size decreases to 10.48 nm, and further with the integration of Nb_2_C MXene, the ternary composite results in a crystallite size of 7.94 nm. This decrease in crystallite size during composite formulation can be attributed to lattice mismatch induced during composite formation, interfacial strain, and growth inhibition, which broaden XRD peaks and limit crystallite growth.^43^ Also, smaller crystallite-size composites possess higher specific surface area, which improves the catalytic activity of the material. Since dislocation density has an inverse relationship with crystallite size, it increases from 34.21 × 10^–4^ nm for pure ZnO to 91.04 × 10^–4^ nm for the binary hybrid, and reaches 158.44 × 10^–4^ nm in the ternary composite. This indicates a higher number of active surface sites and better charge separation.^44^ Furthermore, a clear upward trend is observed in the microstrain, which increases from 6.76 × 10^–3^ for pristine ZnO to 10.99 × 10^–3^ in the binary hybrid, and 14.52 × 10^–3^ in the ternary composite. This pronounced increment in microstrain is direct evidence of lattice distortion and stress induced at the multicomponent heterojunctions, which inherently alter the local electronic band structure and facilitate faster charge-transfer kinetics at the catalyst surface.^45^

FTIR spectroscopy was employed to further elucidate the chemical composition and the interfacial interactions within the binary and ternary composites (Figure 1b). The peaks around 3400 cm^–1^ and 1640 cm^–1^ represent O–H stretching and H–O–H bending, respectively, due to the presence of moisture in the tested material.^46^ The multiple peaks from 420 to 550 cm^–1^ denote Zn–O stretching and Zn–O–Zn bending, confirming the formation of MOF-derived ZnO.^47^ Furthermore, the peak at 1600 cm^–1^ is due to carboxylate COO^–^ stretching arising from a small amount of non-decomposed ZIF-8 residues.^48^ For the NiCo_2_O_4_ samples, peaks at 555 cm^–1^ and 650 cm^–1^ are associated with the stretching and bending vibrations of Ni–O and Co–O bonds.^49^ The additional peak at 1385 cm^–1^ might be due to N–H vibrations from urea residue.^50^ For Nb_2_C MXene, the characteristic peaks of Nb–C bonds were found at 520 and 550 cm^–1^.^51,52^ For the binary composite (ZnO/NiCo_2_O_4_), all peaks of the parent materials are present without any shift in position. However, for the ternary composite (ZnO/NiCo_2_O_4_/Nb_2_C), there is a shift in the metal–oxygen stretching frequencies of ZnO and NiCo_2_O_4_ after forming the composite, indicating a strong interfacial interaction between the oxides and the Nb_2_C MXene sheets.

The optical absorption characteristics of the synthesized materials were investigated using UV-visible spectroscopy (UV-vis). As shown in Figure 2a, pure MOF-derived ZnO exhibits an absorption band in the UV region and extends toward the visible region. Upon the integration of NiCo_2_O_4_ and Nb_2_C MXene, the absorption profile of the binary and ternary composites demonstrates a significant red shift toward the visible light region up to 580 nm. This spectral shift reflects the altered electronic energy states and interfacial electronic coupling between the ZnO nanostructures, the spinel NiCo_2_O_4_ phase, and the high-conductivity Nb_2_C MXene sheets.^53^ The bandgap (Eg) is calculated using the Tauc plot and shows that MOF-derived ZnO has a bandgap of 2.1 eV. The pristine ZnO typically exhibits a wide direct bandgap around 3.3 eV in the UV region.^54^ MOF-derived ZnO synthesized here shows a reduced optical bandgap due to the presence of in situ carbonaceous residues acting as dopants and intrinsic oxygen vacancies.^55^ Compared to MOF-derived ZnO, for binary and tertiary composites, there is a subsequent reduction in bandgap by 0.1 and 0.2 eV, respectively, due to the formation of heterojunctions.^56^

**Figure 2:**
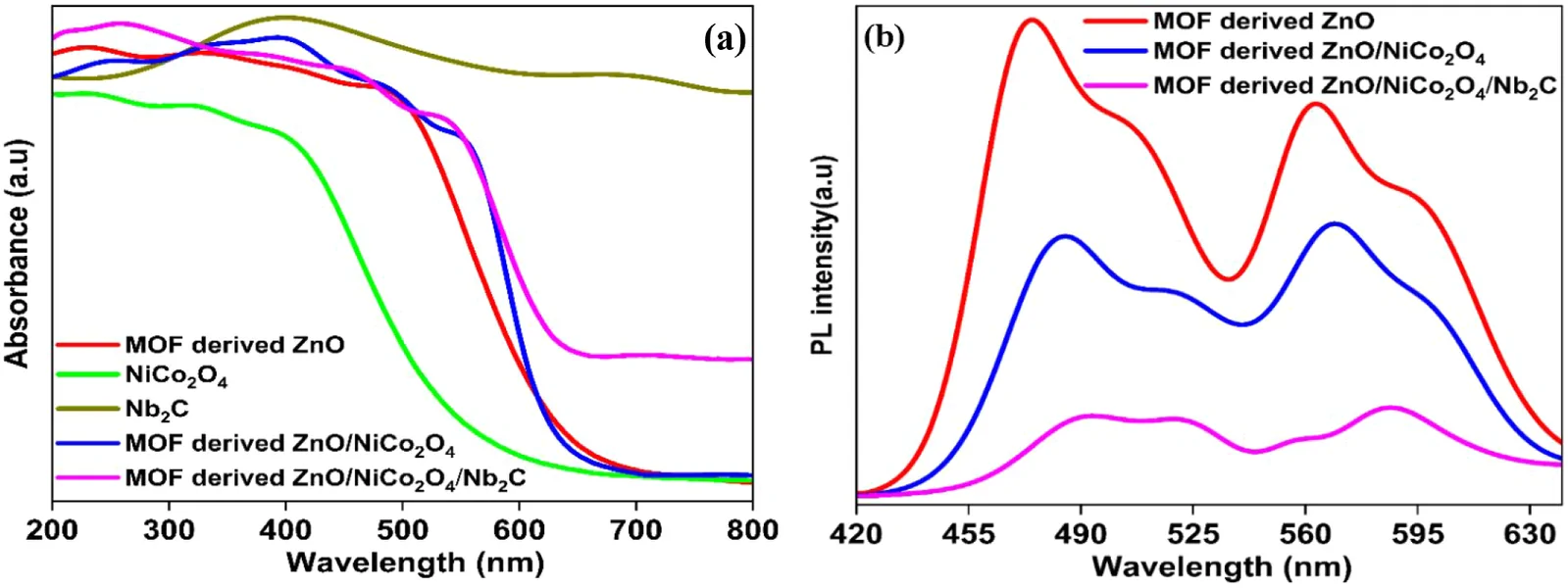
(a) UV–visible spectrum of the synthesized CE materials; (b) photoluminescence spectrum of the synthesized CE materials.

Photoluminescence (PL) spectroscopy (Figure 2b) was used to further examine the defect structures and electronic state interactions. The pristine ZnO exhibits two emission bands observed between 430 and 610 nm, associated with intrinsic structural defects and oxygen vacancies.^57^ The addition of NiCo_2_O_4_ and Nb_2_C leads to significant attenuation of PL intensity. This PL quenching serves as qualitative structural evidence of non-radiative decay pathways and electronic interactions across the heterojunction interface, confirming effective material integration.

The morphological characteristics of the synthesized counter electrode materials were systematically investigated using Field Emission Scanning Electron Microscopy (FESEM) and High-Resolution Transmission Electron Microscopy (HRTEM). The MOF-derived ZnO formed as hexagonal cylindrical morphology with a diameter of 1 μm (Figure 3a). In Figure 3b the NiCo_2_O_4_ phase is shown as spherical nanoparticles with dimensions less than 100 nm, and Figure 3c shows the two-dimensional (2D) sheet-like layered structure of the Nb_2_C MXene. FESEM and HRTEM images in Figure 3d and 3f respectively illustrate the integration of these components into a hierarchical ternary architecture. The analysis confirms that the three architectures were well dispersed, resulting in a complex multi nanostructure. The elemental composition and spatial distribution of the synthesized ZnO/NiCo_2_O_4_/Nb_2_C composite were investigated via Energy-Dispersive X-ray (EDX) spectroscopy and elemental mapping. The EDX spectrum (Figure 3e) confirms the presence of Zn, Ni, Co, Nb, C, and O, with peak intensities corresponding to the constituent phases of the ternary system. The absence of additional peaks in the EDX spectrum further validates the high chemical purity of the synthesized material, ensuring that no extraneous contaminants were introduced during the composite fabrication process.

**Figure 3:**
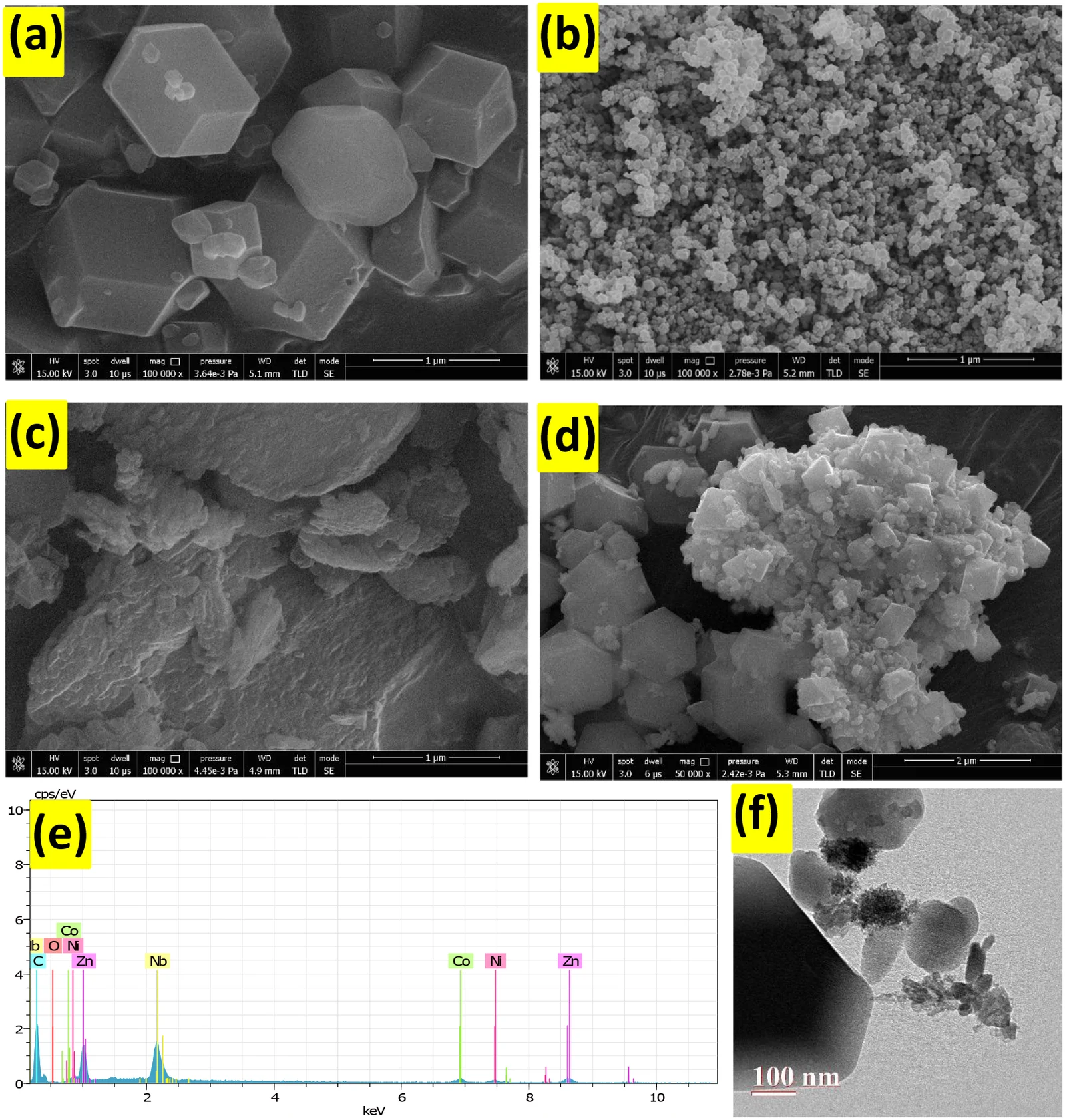
SEM images: (a) MOF-derived ZnO, (b) NiCo_2_O_4_ (c) Nb_2_C MXene, (d) ZnO/NiCo_2_O_4_/Nb_2_C, (e) EDX spectrum of ZnO/NiCo_2_O_4_/Nb_2_C, and (f) HRTEM of ZnO/NiCo_2_O_4_/Nb_2_C.

The surface electronic structure and elemental composition of the synthesized ZnO/MOF/NiCo_2_O_4_/Nb_2_C composites were analyzed by X-ray photoelectron spectroscopy (XPS), and the resultant spectra are shown in Figure 4a–h. The survey spectrum of Figure 4a confirms the coexistence of Zn, Ni, Co, Nb, O, N, and C elements, signifying the successful formation of a hybrid architecture. The intense C 1s and N 1s signals indicate the presence of a nitrogen-enriched carbonaceous matrix, resulting from the parent MOF structure, while the characteristic Zn 2p, Ni 2p, Co 2p, O 1s, and Nb 3d peaks substantiate the successful integration of ZnO, spinel NiCo_2_O_4_, and Nb_2_C phases.

**Figure 4:**
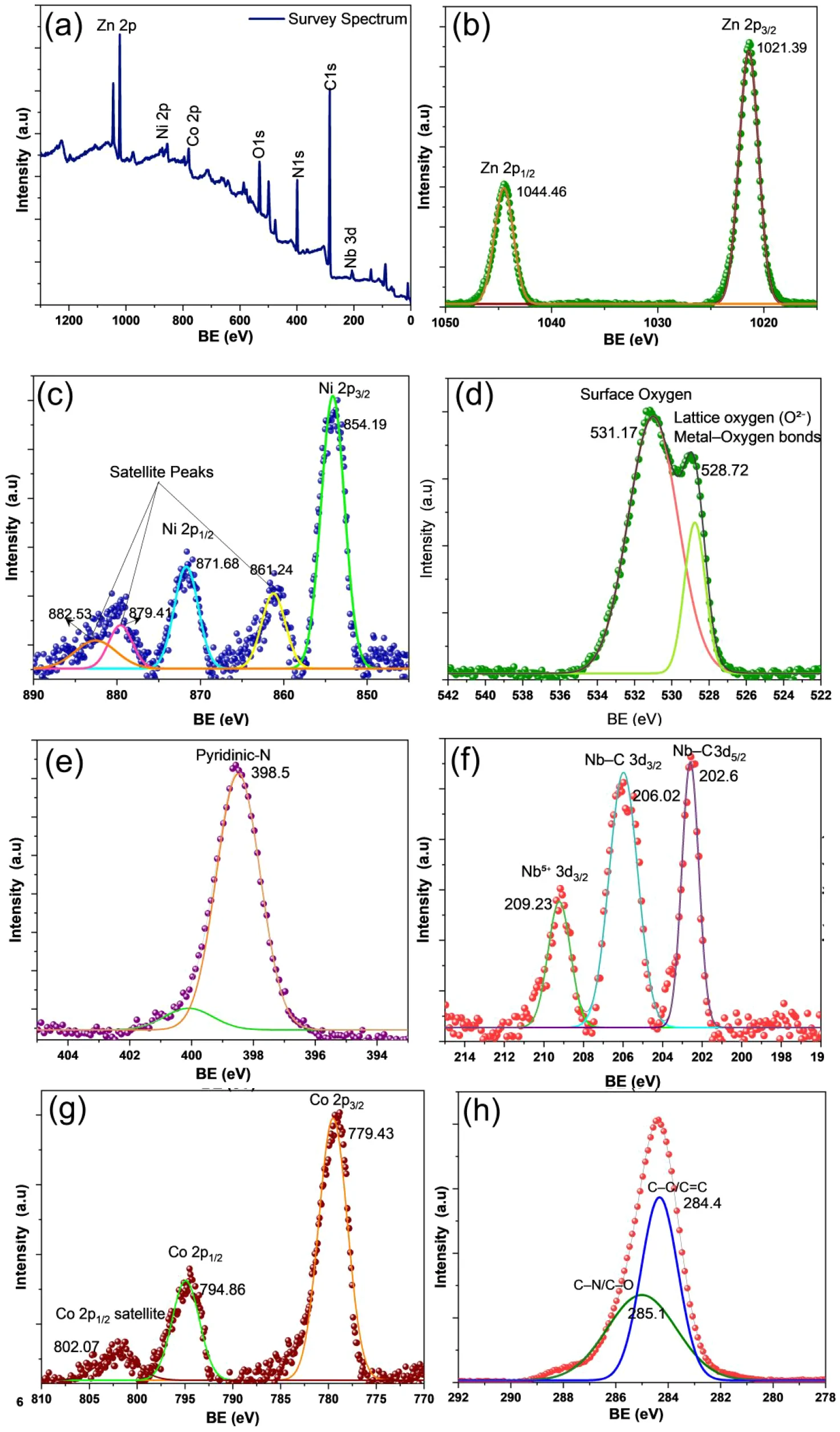
XPS spectra of the ZnO/MOF/NiCo_2_O_4_/Nb_2_C composite: (a) survey spectrum and high-resolution spectra of (b) Zn 2p, (c) Ni 2p, (d) O 1s, (e) N 1s, (f) Nb 3d, (g) Co 2p, and (h) C 1s.

The high-resolution Zn 2p spectrum (Figure 4b) exhibits two distinct spin–orbit doublets located at 1021.39 and 1044.46 eV, corresponding to Zn 2p_3_/_2_ and Zn 2p_1_/_2_. The spin–orbit splitting energy of 23.07 eV corresponds to the characteristic Zn^2+^ oxidation state in hexagonal ZnO, confirming the oxidation of Zn species and formation of the ZnO crystal lattice.^58–60^ The absence of satellite peaks further eliminates the presence of metallic Zn.

The Ni 2p spectrum displayed in Figure 4c shows two major peaks centered at 854.19 and 871.68 eV, corresponding to Ni 2p_3_/_2_ and Ni 2p_1_/_2_. Further, shake-up satellite peaks at 861.24 and 879.41 eV were observed. These intense satellite peaks are specific to Ni^2+^ species. The asymmetric broadening of the main peaks suggests the coexistence of Ni^3+^ species within the spinel framework. Such mixed-valence Ni^2+^/Ni^3+^ states are characteristic of inverse spinel NiCo_2_O_4_ and are known to stimulate rapid Faradaic redox reactions through enhanced electron hopping between adjacent metal centers, thereby enhancing electrochemical kinetics and electrical conductivity.

The deconvoluted O 1s spectrum of Figure 4d consists of two distinct oxygen environments centered at 528.72 and 531.17 eV. The low-binding-energy peak at 528.72 eV is ascribed to lattice oxygen (O^2–^) coordinated with Zn, Ni, and Co atoms within the metal-oxide framework. On the other hand, the higher-binding-energy peak at 531.17 eV corresponds to surface chemisorbed oxygen species, hydroxyl groups, and defect-associated oxygen vacancies.^61^ The high relative area of this peak confirms that the MOF route successfully induced abundant surface defect sites. The relatively high intensity of the defect-related oxygen peak indicates the presence of abundant oxygen-deficient sites during thermal treatment and heterostructure formation. These oxygen vacancies can efficiently curb the local electronic structure, lower charge-transfer resistance, and offer additional electrochemically active sites, thereby facilitating electrolyte-ion diffusion and interfacial redox reactions.

High-resolution N 1s spectrum (Figure 4), in which the peak located at 398.5 eV corresponds to pyridinic nitrogen species, which originates from the MOF-derived nitrogen-doped carbon framework and is usually located at defect sites. This can considerably enhance the electrical conductivity by altering the electron density distribution of adjacent carbon atoms while creating additional active sites for charge storage and surface redox reactions. The incorporated nitrogen heteroatoms can increase the surface wettability and strengthen interfacial interactions between carbon and metal oxide.

The Co 2p spectrum given in Figure 4g exhibits specific Co 2p_3_/_2_ and Co 2p_1_/_2_ peaks centered at 779.43 and 794.86 eV, along with a shake-up satellite feature at 802.07 eV. The obtained lower binding-energy component is associated with Co^3+^ species, whereas the satellite peak observed confirms the simultaneous presence of Co^2+^ ions within the spinel lattice.^62^ The coexistence of Co^2+^/Co^3+^ redox couples corresponds to the spinel NiCo_2_O_4_ and is beneficial for improving the electronic conductivity through intervalence charge-transfer mechanisms. The mixed-valence states contribute to reversible redox transitions and improved electrochemical activity during the charge-transfer process.

The Nb 3d spectrum shown in Figure 4f establishes the effective incorporation of niobium carbide into the composite structure. The specific peaks located at 202.6 and 206.02 eV correspond to Nb–C 3d_5_/_2_ and Nb–C 3d_3_/_2_ spin–orbit components, confirming the formation of Nb_2_C.^63^ The observed spin–orbit splitting reported in the literature for transition-metal carbides. Also, a small peak located at 209.23 eV is ascribed to Nb^5+^ species originating from the oxidation of Nb_2_C upon exposure to air. The coexistence of conductive Nb_2_C and thin surface niobium oxide layers can provide synergistic advantages, where Nb_2_C contributes high electrical conductivity and rapid electron transport, while the surface oxide species may introduce additional catalytic active sites.

The C 1s spectrum displayed in Figure 4h is deconvoluted into two major peaks at 284.4 and 285.1 eV, corresponding to graphitic C–C/C=C and C–N/C–O bonding, respectively. The dominant graphitic carbon peak indicates the formation of conductive carbon domains derived from the carbonization of the MOF structure, which facilitates the interconnected electron-transport network throughout the composite. Also, the nitrogen incorporation into the carbon matrix provides additional defect-rich active sites that enhance the interfacial charge-transfer process.

The XPS results confirm the successful formation of the ZnO/MOF/NiCo_2_O_4_/Nb_2_C heterostructure with mixed-valence metal species, conductive Nb_2_C phases, nitrogen-doped carbon, and oxygen-vacancy-rich defect sites. The synergistic interaction among these components can facilitate a facile electron–ion transport pathway. Also, the structure is capable of enhancing redox kinetics and improving the overall electrochemical characteristics of the composite electrode. Furthermore, a slight shift in the binding energies of the metal oxides ZnO and NiCo_2_O_4_ is observed in the composite compared to the literature data for the pristine form.^64,65^ This shift indicates a redistribution of electron density at the heterointerfaces, providing strong evidence of a heterostructure with electronic coupling between the metal oxides and the Nb_2_C MXene sheets.^66^ This synergistic electronic interaction facilitates more efficient charge-carrier separation and migration, which contributes to the enhanced power conversion efficiency in the DSSC measurements.

The photovoltaic performance of the fabricated dye-sensitized solar cells (DSSCs) using the synthesized counter electrodes (CEs) was evaluated, and the results are presented in Figure 5 and Table 2. The device fabricated using the MOF-derived ZnO CE yielded an open-circuit voltage *V*_oc_ of 0.56 V and a short-circuit current density *J*_sc_ of 7.51 mA/cm^2^, resulting in a power conversion efficiency of 3.11%. Similarly, the NiCo_2_O_4_ and Nb_2_C CE-based DSSCs have obtained efficiencies of 4.56% and 2.51%, respectively. In comparison, the binary and ternary composite counter electrodes demonstrated substantially superior performance. MOF ZnO/NiCo_2_O_4_ obtained a *V*_oc_ of 0.65 V and a *J*_sc_ of 13.58 mA/cm^2^, and MOF-derived ZnO/NiCo__2__O__4__/Nb__2__C obtained a *V*_oc_ of 0.71 V and a *J*_sc_ of 16.53 mA/cm^2^, which correspond to enhanced efficiencies of 6.15% and 8.08%, respectively. This represents a more than 2.6 times increase in efficiency compared with the pristine ZnO counterpart, highlighting the efficacy of the ternary architecture. Also, when compared to the standard Pt counter-electrode DSSC, there is a slight improvement in the current density and a very small reduction in fill factor. However, the overall efficiency of the fabricated Pt DSSC (7.97%) remains very close to that of the MOF-derived ZnO/NiCo__2__O__4__/Nb__2__C DSSC (8.08%).

**Table 2:** Photovoltaic Performance of the Fabricated DSSCs

**Counter Electrodes**	***J***__**sc**__ **(mA cm** ^^**–2**^^)	***V***__**oc**__ **(V)**	**FF**	**PCE %**
MOF-derived ZnO	7.512	0.56	0.738	3.108
NiCo_2_O_4_	10.530	0.62	0.699	4.569
Nb_2_C	5.569	0.62	0.728	2.517
MOF-derived ZnO/NiCo_2_O_4_	13.580	0.65	0.697	6.157
MOF-derived ZnO/NiCo__2__O__4__/Nb__2__C	16.531	0.71	0.688	8.080
Pt	15.620	0.71	0.71	7.970

**Figure 5:**
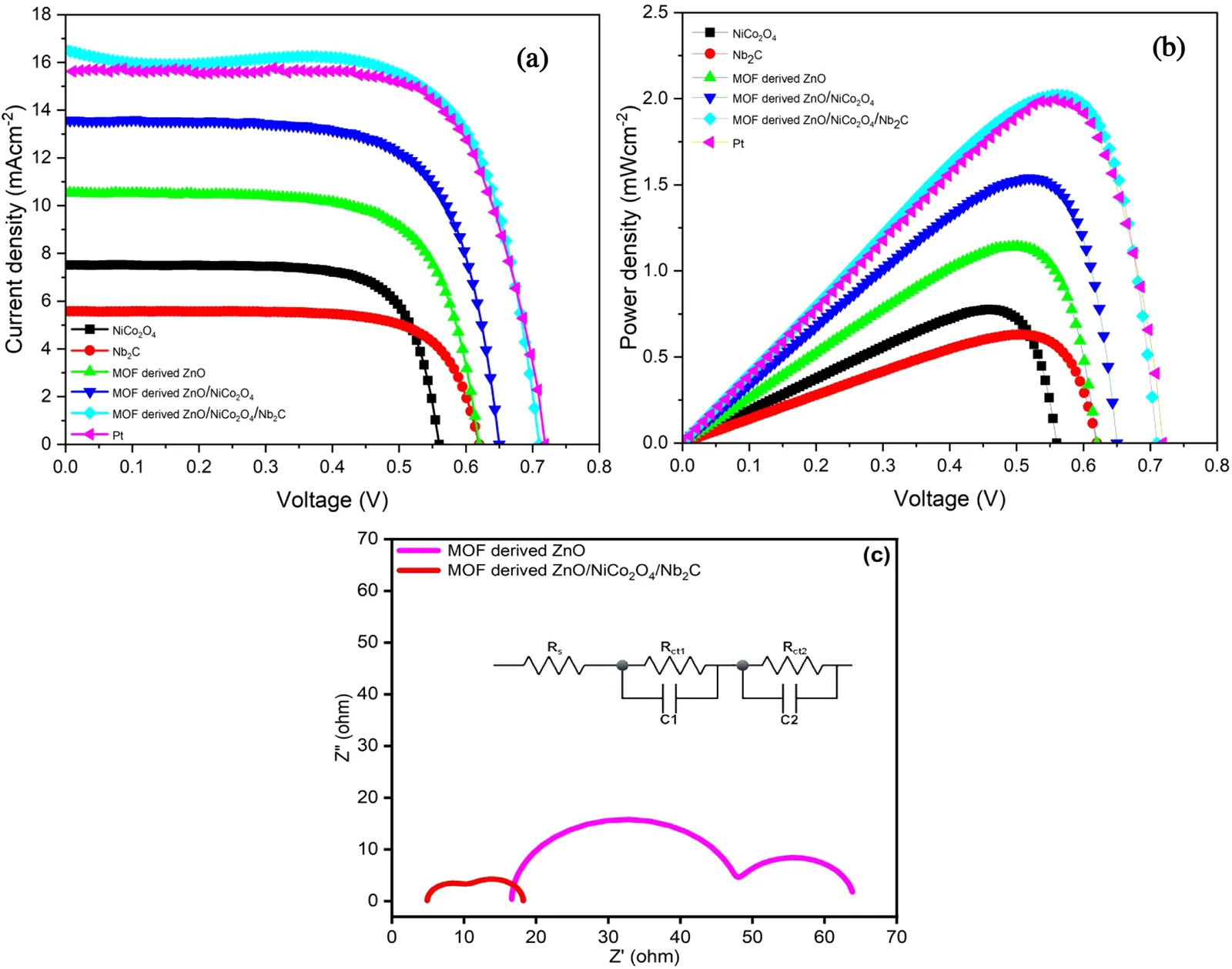
DSSC performance of the fabricated CEs: (a) photocurrent density versus voltage (*J*–*V*), (b) power density versus voltage (*P*–*V*), and (c) Nyquist plot with the equivalent circuit used to fit the impedance curve.

In previous studies ZnO/C CE-based DSSCs have reported an efficiency of 6.7%.^67^ Also, ZnO is used with PEDOT:PSS composite CEs to increase catalytic activity and improve the DSSC PCE.^68^ Similarly, NiCo_2_O_4_ is a widely utilized and promising Pt-free CE that delivers high catalytic activity toward I^–^/I_3_^–^ reduction, good conductivity, and significant cost advantages over Pt. Wang et al. achieved 6.27% using a NiCo_2_O_4_ carbon black composite, reporting improved electrocatalytic activity for triiodide (I_3_^–^) reduction and structural stability afforded by carbon-based synergistic effects.^69^ Similarly, studies have explored NiCo_2_S_4_ as a CE, yielding efficiencies up to 6.53%.^70^ In the present study, the superior efficiencies of 6.15% and 8.08% achieved by the binary and tertiary composite CEs, respectively, can be attributed to several synergistic factors. The hierarchical integration of NiCo_2_O_4_ and ZnO effectively optimizes the catalytic active site distribution, minimizing charge transfer resistance. Furthermore, the introduction of Nb_2_C MXene significantly enhances the overall electrical conductivity and accelerates electron transfer kinetics at the electrolyte-electrode interface. The substantial improvement in *V*_oc_ and *J*_sc_ demonstrates that the ternary heterojunction effectively modulates the electrochemical potential, confirming this material as a high-performance candidate for DSSC applications. Table 3 compares the photovoltaic performance of DSSCs with Pt-free electrodes published with the present study.

**Table 3:** Comparative Analysis of the Photovoltaic Performance of DSSCs with Pt-Free Electrodes

**Counter Electrode Material**	**Power Conversion Efficiency (PCE, %)**	**Refs**
NiCo_2_O_4_ with carbon black	6.27	^69^
NiCo_2_S_4_	6.53	^70^
Co-NiO	5.01	^71^
NiCo_2_O_4_@RGO nanobelts	6.17	^72^
Ti_3_C_2_T_*x*_ MXene	8.68	^34^
MXene/NiCo_2_S_4_	8.76	^73^
MXene–graphene	6.4	^35^
Ti_3_C_2_T_*x*_–PEDOT nanofibers	7.1	^74^
CNTs/Ti_3_C_2_T_*x*_	5.83	^75^
V_2_C MXene/TiO_2_	1.62	^76^
Ti_3_C_2_T_*x*_–polythiophene	5.83	^77^
MOF derived ZnO	3.11	This work
MOF derived ZnO/NiCo_2_O_4_	6.15	This work
MOF derived ZnO/NiCo_2_O_4_/Nb_2_C	8.08	This work

To elucidate the charge-transfer kinetics at the counter electrode/electrolyte interface, electrochemical impedance spectroscopy (EIS) measurements were conducted. The Nyquist plots are presented, and an equivalent circuit was fitted (Figure 5c). *R*_s_ corresponds to the overall ohmic resistance, including the resistance of the FTO substrate and the contact resistance of the coating. *R*_ct1_ and *C*_1_ represent the charge-transfer resistance and double-layer capacitance at the counter electrode/electrolyte interface, where the reduction of triiodide (I_3_^–^) takes place. *R*_ct2_ and *C*_2_ are the charge-transfer resistance and capacitance at the photoanode/electrolyte interface, which are associated with the transfer of electrons between the photoanode and the electrolyte.

The device utilizing the MOF-derived ZnO counter electrode exhibited a relatively high *R*_ct_ (*R*_ct1_ + *R*_ct2_) of 47.24 Ω, indicating slower catalytic kinetics for the triiodide (I_3_^–^) reduction reaction. In contrast, the ternary ZnO/NiCo_2_O_4_/Nb_2_C composite counter electrode displayed a significantly reduced *R*_ct_ of 13.3 Ω. Specifically, *R*_ct1_ associated with the high-frequency charge transfer at the counter electrode/electrolyte interface reduces from 31.38 Ω to 5.65 Ω, indicates easier electron transfer at the CE/electrolyte interface. Furthermore, the series resistance (*R*_s_) decreases significantly from 16.6 Ω for pristine MOF-derived ZnO to 4.89 Ω for the tertiary composite. This reduction confirms the enhanced electrical conductivity due to the incorporation of the highly conductive Nb_2_C MXene network. The EIS characteristics are summarized in Table 4. This drastic decrease demonstrates the enhanced electrocatalytic activity of the composite. The substantial reduction in resistance confirms that the high-conductivity Nb_2_C MXene backbone and the synergistic integration of NiCo_2_O_4_ effectively facilitate rapid electron transfer at the interface, thereby ultimately enhancing the overall power conversion efficiency of the DSSC.

**Table 4:** EIS Characteristics of the Fabricated DSSCs

**Counter Electrodes**	***R***_**s**_ (**Ω)**	***R***_**ct‑1**_ **(Ω)**	***R***_**ct‑2**_ **(Ω)**
MOF derived ZnO	16.6	31.38	15.86
MOF derived ZnO/NiCo__2__O__4__/Nb__2__C	4.89	5.65	7.65

To further evaluate the catalytic activity and charge-transfer resistance, cyclic voltammetry was performed and is shown in Figure 6a and 6b. Cyclic voltammetry showed two redox couples for all prepared samples, where the first and DSSC-relevant pair corresponds to (I_3_^–^ + 2e^–^→3I^–^) at *E*_pa_ and (I_3_^–^ + 2e^–^ → 3I^–^) at *E*_pc_, while the second pair is associated with (2I_3_^–^ → 3I_2_ + 2e^–^) and its reverse reaction. The peak separation was calculated as Δ*E*_p_ = *E*_pa_ – *E*_pc_. MOF-derived ZnO exhibited *E*_pa_ =0.61902 V and *E*_pc_ = 0.39672 V, giving Δ*E*_p_ =0.22230 V; NiCo__2__O__4__ and Nb__2__C showed Δ*E*_p_ =0.56771 V and Δ*E*_p_ = 0.40812 V, respectively. MOF-derived ZnO/NiCo__2__O__4__ exhibited Δ*E*_p_ =0.08265 V and the tertiary composite MOF-derived ZnO/NiCo__2__O__4__/Nb__2__C showed *E*_pa_ = 0.59565 V and *E*_pc_ = 0.61218 V, resulting in the smallest absolute separation of Δ*E*_p_ =0.01653 V. The substantially smaller Δ*E*_p_ of MOF-derived ZnO/NiCo__2__O__4__/Nb__2__C indicates lower electrochemical polarization, greater redox reversibility, and faster interfacial electron-transfer kinetics for triiodide reduction.^78^ Similarly, the cathodic peak current density (*J*_pc_) and the area under the CV curve were evaluated for all tests, and the highest *J*_pc_ (0.85 mA cm^–2^) and area are recorded for the MOF-derived ZnO/NiCo__2__O__4__/Nb__2__C composite, indicating a higher reduction rate, greater density of active catalytic sites, and enhanced overall device current density.^79^ This confirms that the tertiary composite has a higher density of catalytically active sites, minimizing charge-transfer resistance, thereby increasing the overall performance of the fabricated DSSCs.

**Figure 6:**
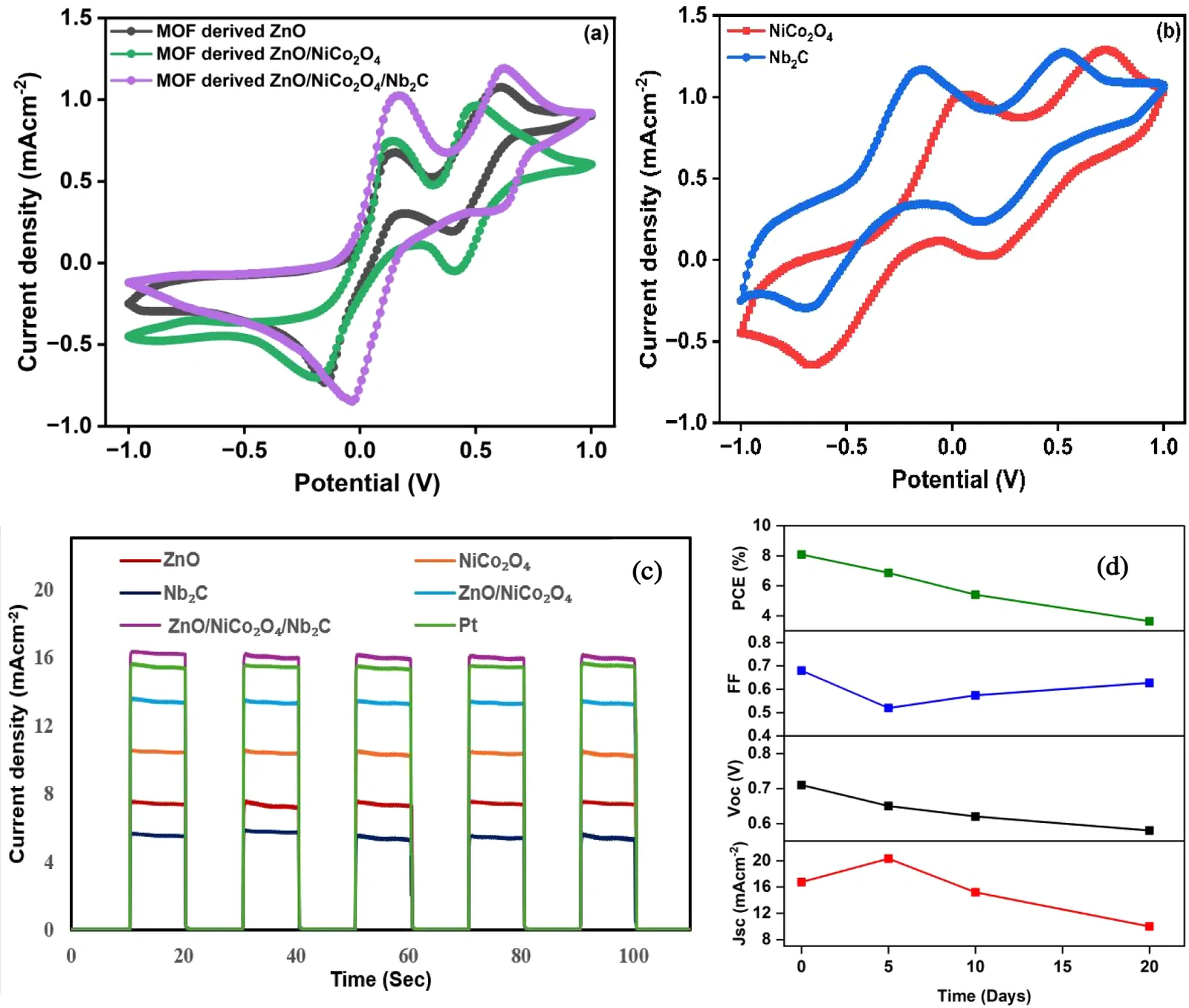
Cyclic voltammetry analysis (a) and (b), photocurrent response (c), and long-term stability analysis (d) of the fabricated DSSCs.

To evaluate the stability of the prepared DSSCs, transient photocurrent density and long-term stability tests were performed. The transient photocurrent density for the prepared materials was evaluated for 5 cycles (10 s off/10 s on) and the results are shown in Figure 6c. In the absence of light, photocurrent measurements were zero. Also, the photocurrent density remained stable for all materials throughout the 5 cycles. A long-term stability test for the ternary composite DSSC was performed for up to 20 days, during which measurements were taken at regular intervals, and the device was kept in the dark after testing. The results are shown in Figure 6d. It can be seen that all PV performance parameters dropped slightly during the initial days. The DSSC cells were stable, and the efficiency dropped by only 1.2% in 5 days. However, there is a very high drop in *J*_sc_ after 8 days due to thermal stress and electrolyte degradation and leakage.^80^ Subsequently, the efficiency of the DSSC was also reduced from 8.08% to 3.64%.

## 4 Conclusion

The study successfully demonstrates the synthesis of a high-performance, platinum-free counter electrode utilizing a ternary MOF-derived ZnO/NiCo_2_O_4_/Nb_2_C MXene composite via a hydrothermal route. Structural and morphological characterizations through XRD, FESEM, and HRTEM confirmed the successful integration of hexagonal ZnO cylinders, spherical NiCo_2_O_4_ nanoparticles, and conductive 2D layered Nb_2_C MXene sheets, forming a robust hierarchical architecture. The optimized ternary composite achieved a remarkable power conversion efficiency (PCE) of 8.08%, which represents a significant 2.6-fold improvement over pristine MOF-derived ZnO and serves as a competitive alternative to conventional Pt-based electrodes. This enhanced performance is primarily attributed to the abundant active sites in the tertiary composite for the triiodide (I_3_^–^) reduction reaction, as confirmed by cyclic voltammetry analysis. Also, the enhanced charge kinetics from the metallic conductivity of the Nb2C MXene backbone facilitated rapid electron transport, effectively reducing the charge-transfer resistance (Rct) to 0.71 Ω. These results suggest that the ZnO/NiCo_2_O_4_/Nb_2_C MXene architecture provides a viable pathway toward the development of sustainable, stable, cost-effective, and efficient DSSC technologies.

## Data Availability

The raw/processed data required to reproduce these findings are available from the corresponding author on request.

## Abbreviations

DSSC: Dye-sensitized solar cell
CE: counter electrode
XRD: X-ray diffraction
FTIR: Fourier transform infrared spectroscopy
UV-Vis: UV–Visible spectroscopy
PL: photoluminescence
FESEM: field-emission scanning electron microscopy
EDX: energy-dispersive X-ray spectroscopy
HRTEM: high-resolution transmission electron microscope
*V*_oc_: open-circuit voltage
*J*_sc_: short-circuit current density
FF: fill factor
PCE: power conversion efficiency
Pin: input power
*V*_mp_: maximum voltage
*J*_mp_: maximum current density
